# Agricultural drought-driven mechanism of coupled climate and human activities in the karst basin of southern China

**DOI:** 10.1038/s41598-024-62027-w

**Published:** 2024-05-27

**Authors:** Shan Pan, Zhonghua He, Xiaolin Gu, Mingjin Xu, Lihui Chen, Shuping Yang, Hongmei Tan

**Affiliations:** 1 School of Geography and Environmental Science, Guizhou Normal University, Guiyang, 550001 China; 2 National Engineering Technology Research Center for Karst Rocky Desertification Control, Guizhou Normal University, Guiyang, 550001 China; 3 Guizhou Key Laboratory of Remote Sensing Application of Mountain Resources and Environment, Guiyang, 550001 China; 4 Guizhou Hydrology and Water Resources Bureau, Guiyang, 550002 China

**Keywords:** Driving mechanisms, Agricultural drought, Climate change, Human activities, Southern China, Climate sciences, Environmental sciences, Environmental social sciences, Hydrology, Planetary science

## Abstract

Timely and accurate agricultural drought monitoring and drought-driven mechanism analysis in karst basins in the context of global warming are highly important for drought disaster monitoring and sustainable ecological development in a basin. In this study, based on MODIS data, meteorological and topographic data and land use data from 2001 to 2020, we used the Sen slope, the Mann–Kendall test and a geographic detector to explore the driving mechanisms of agricultural drought caused by climate change and human activities in the karst basin of southern China from 2001 to 2020. The results showed that (1) the spatial distribution of the TVDI in the karst basin in southern China has obvious regional characteristics, showing a decreasing trend from west to east. (2) According to the interannual trend of drought, the degree of drought in the South China karst basin exhibited a weakening trend over the last 20 years, with the most severe drought occurring in 2003. Regarding the seasonal change in the TVDI, drought in spring, summer and autumn exhibited a decreasing trend, while that in winter exhibited an increasing trend, and the drought intensity decreased in the following order: spring (0.58) > autumn (0.53) > summer (0.5) > winter (0.48). (3) Single-factor detection the results showed that rainfall, temperature and elevation were the main factors driving aridification in the study area; multifactor coupling (mean) drove drought in descending order: rainfall (q = 0.424) > temperature (q = 0.340) > elevation (q = 0.219) > land use (q = 0.188) > population density (q = 0.061) > slope (q = 0.057). Therefore, revealing the mechanism of agricultural drought in karst basins through the study of this paper has important theoretical significance and provides technical guidance for drought relief in karst areas.

## Introduction

Agricultural drought is a phenomenon in which an imbalance between water balance and water deficit occurs in vegetation or crops due to external environmental factors, affecting the normal growth and development of vegetation or crops and leading to plant wilting or death^1^. Agricultural drought usually causes a series of social and environmental problems and enormous economic losses^2^. In the twenty-first century, global warming has become a major environmental problem. In the context of global warming, the global trend of drought is becoming increasingly serious. For example, in 2021, statistics from the United Nations Office for Disaster Risk Reduction showed that the average annual loss of agricultural drought in the United States alone reached 6.4 billion US dollars, and the average annual loss in Europe reached 9 billion US dollars^3^. In 2006–2017, the direct economic losses caused by drought in China averaged 88.230 billion US dollars annually, and the area of the crop affected was 169 million hm^2^^4^. Therefore, highly important to research the characteristics and mechanisms of agricultural drought evolution in river basins for the purposes of scientific and reasonable drought relief, drought dynamics monitoring, risk assessment and emergency program development.

Agricultural drought, as one of the important impacts of global climate change, has become a serious hindrance to agricultural production worldwide. The increased frequency of extreme meteorological events also exposes agriculture to greater uncertainty^5^, and climate change due to warming, as well as the impacts of human activities on land use and water resource management, has combined to contribute to the occurrence and exacerbation of agricultural droughts. Rainfall is now distributed more unevenly and in smaller amounts due to changes in global precipitation patterns brought on by climate change. This has exacerbated agricultural droughts and enhanced evaporation in conjunction with high temperatures, placing farmers at risk of water shortages^6^. The impact of human activities on agricultural drought is mainly reflected in land use, water management and agricultural practices. With accelerated urbanization and industrialization, a large amount of agricultural land has been converted to industrial land or urban construction land, leading to reduced water use for agriculture and land degradation^7^. Irrational water resource management and over-exploitation of groundwater have also led to water scarcity and declining water quality; and large-scale irrigation and inappropriate tillage in agricultural practices have also exacerbated soil moisture evaporation and the risk of drought in agricultural land^8^. Agricultural drought threatens food security and sustainable agricultural development, prompting an in-depth study of the relationship between climatic factors and human activities on agricultural drought to understand the links and impacts between the two, which can help to formulate effective policies and measures to improve the sustainability of agricultural production^9^, especially as the ecological environment of karst regions is extremely fragile^10^. Understanding the impacts of climatic factors and human activities on agricultural drought can help governments and agricultural managers develop appropriate risk management measures to reduce the economic and ecological impacts of agricultural drought^11^.

Agricultural drought is a drought problem triggered by soil moisture deficits due to prolonged rainfall deficits. Currently, an important indicator in the quantitative description of meteorological drought is the Palmer drought severity index (PDSI), which has the advantages of integrating rainfall, soil moisture, runoff, and potential evapotranspiration^12^ and has a clear physical mechanism that can be used to monitor the long-term evolution of drought; thus, the PDSI has been widely used in drought monitoring^13–15^. Compared with meteorological drought indicators, the soil moisture deficit indicator called the soil moisture index (SMI), is more immediate and accurate for monitoring and assessing agricultural drought^16–18^. Vegetation indices, including the normalized difference vegetation index (NDVI), vegetation condition index (VCI) and vegetation health index (VHI) have been used to characterize agricultural droughts^19,20^ and have been widely applied in global and regional agricultural monitoring^21,22^. The temperature vegetation drought index (TVDI), a drought monitoring index based on an empirical parameterization of the land surface temperature (LST–NDVI) space, has been widely implemented in a variety of ecosystems worldwide. Compared with other agricultural drought indicators, such as the NDVI, which has a lag in soil moisture monitoring, and the temperature condition index (TCI), which is not applicable to complex terrain monitoring^23^, the combination of the two constructed TVDIs compensates for the shortcomings of each; and removes the effects of different vegetation cover types, making the TVDI more suitable for agricultural drought monitoring in any vegetation cover area. For example, Sandholt^24^ proposed NDVI-LST eigenspaces using the simplified temperature vegetation drought index (TVDI), which is based on the simplified NDVI-LST triangular and trapezoidal eigenspaces of remotely sensed data, and these eigenspaces can be used to better monitor drought conditions in arid and semiarid areas. Patel^25^ and Chen^26^ used the TVDI to infer the potential of soil moisture status and to characterize droughts spatially and temporally. Du^27^ used two distinct modeling techniques for the purpose of tracking drought in Ningxia, a semiarid area in China. They employed both the conventional TVDI as a substitute for the LST and the difference between diurnal and nocturnal surface temperatures (ΔLST), which was computed using TVDIm. According to the study, TVDI was shown to have a larger magnitude than TVDIm. This could result in low TVDI values indicating drought conditions in dry years and high TVDI values indicating drought conditions in normal years. Chen^28^ and Li^29^ used the TVDI and standardized precipitation evapotranspiration (SPEI) to assess the drought status in the study area; Liang et al.^30^ used MODIS data as the basis to obtain the TVDI to derive the spatial and temporal distributions of drought in China during 2001–2010; and analysed the relationships between drought and climate factors. Many scholars have used correlation analysis to explore the driving mechanism of agricultural drought^31–33^; Shaban^34^ noted that Lebanon, located in the Mediterranean region, has experienced a drastic decrease in water resources under the influence of climate change and human activities, and this change has led to the occurrence of hydrological drought. Zhang^35^ investigated spatiotemporal variations in drought hazards and driving factors in the Xihaigu region; and found that the driving factors affecting the spatial and temporal changes in drought disasters included climatic, ecological and demographic factors. When exploring regional drought and its influencing factors, the main consideration was meteorological factors such as temperature and precipitation, the influence of topographical factors such as elevation on the distribution of drought was seldom considered, and drought was also affected by human activities in addition to the influence of factors within the system^36^.

Due to the special topographic and hydrological conditions of the karst region, agriculture in this region is more vulnerable to drought, and problems such as land degradation and water resource insufficiency are more prominent; therefore, our purpose in studying agricultural drought in the karst region is to reveal the mechanism of drought impacts on agricultural production, to provide a scientific basis for the development of effective adaptive measures^37^. Agricultural drought studies in karst basins have focused on the spatial and temporal evolution of drought characteristics^38–40^; and have used the agricultural drought index to analyse the spatial and temporal distribution characteristics of drought characteristics (drought intensity, drought frequency, etc.) in the study area. Compared with normal watersheds, karst watersheds not only have many types of media, impure textures, and complex structures but also form different sizes of soluble water under the action of differential erosion or dissolution of soluble water in the form of slots, holes, and pipelines, which provide space and venues for atmospheric precipitation to remain on the surface and underground, resulting in a certain degree of drought mitigation in karst watersheds for agricultural droughts^41^.

In summary, current research on agricultural drought focuses mainly on drought phenomena or results and lacks research on agricultural drought processes and mechanisms^40,42,43^. Therefore, based on MODIS, meteorological and topographic data and land use data from 2001 to 2020, this paper adopted the climate propensity rate to explore the characteristics of climate change in southern China and analysed the characteristics of overall land use change. This paper used Sen slope estimation and the Mann–Kendall test to study the spatial and temporal distributions of agricultural droughts in karst basins in southern China. With the help of geo-detectors, the coupling of climate change and human activities on agricultural drought in southern China was revealed; to provide scientific references for an early warning system of drought in the karst basin in southern China and the formulation of anti-disaster measures in agriculture.

## Overview of the study area

**Figure 1:**
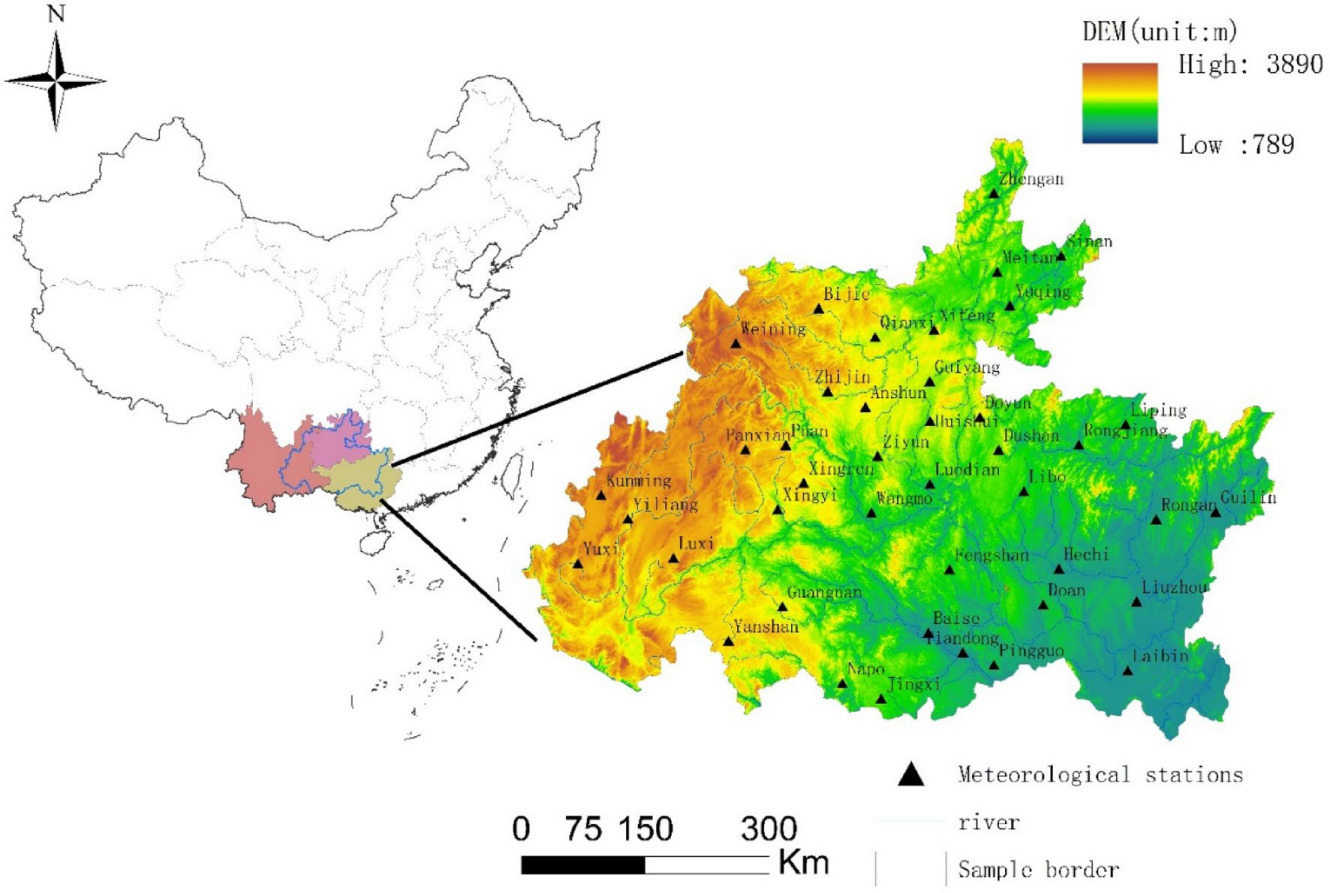
Distribution of major rivers and their meteorological stations in the study area. The map was created with ArcGIS version 10.2 (https://www.arcgis.com/).

The southern China karst basin, centred on Guizhou, Yunnan and Guangxi is a typical distribution area of cone karst, sword karst and tower karst^44^, and this paper used 42 meteorological priorities as the study area in the typical distribution area of karst in southern China (Fig. 1). The study area is located at longitude 101°55′55″ ~ 110°55′45″E, latitude 22°42′57″ ~ 29°13′11″N, with an area of 352,526 km^2^, including most of Guizhou Province (37.97%), southeastern Yunnan Province (25.36%), and northwestern and northern Guangxi Province (36.67%). The terrain is high in the west and low in the east, with plateaus, hills, mountains and basins dominating the landscape, with an average elevation of 848 m. The geomorphological lithology is divided into limestone karst, dolomite karst, gypsum karst and salt karst all of which are soluble; the vegetation is mainly broad-leaved forests, thickets and scrub. The study area is located in the humid climate zone of the northern subtropics and the semihumid climate zone of southern subtropics, with abundant rainfall occurring year round but with an uneven spatial and temporal distribution, with an average rainfall of 1000 ~ 1300 mm and an average annual temperature of 16 ~ 23 °C throughout the area. These special geological and geomorphological types and climatic characteristics cause rocks to gradually dissolve through by water, forming caves and surface landforms with different shapes and forms, mainly stone buds, stone grooves, stone forests, peak forests, drop caves, funnels, karst depressions, caverns, underground rivers and so on.

ArcGIS 10.2 was used to automatically extract basin elevation and hydrological data from a digital elevation model (DEM) with a spatial resolution of 30 m.

## Research data and methodology

### Research data

The data in this study included meteorological data (https://data.cma.cn/), month-by-month temperature data and rainfall data from 42 selected meteorological control stations (24 in Guizhou, 12 in Guangxi, and 6 in Yunnan). ASTER GDEM 30 m data (https://www.gscloud.cn/) were mainly used to extract the basin slope and hydrological characteristics. MODIS data (https://ladsweb.nascom.nasa.gov) were mainly used to invert the surface temperature index (Ts) and normalized difference vegetation index (NDVI), where the LST had a spatial resolution of 1 km and a temporal resolution of 8 day, and the NDVI had a spatial resolution of 1 km and a temporal resolution of 16 day. In addition, land use data (30 m)^45^ and population density data (https://www.resdc.cn/) were used to analyse the land use change and centre of gravity migration in southern China from 2001 to 2020 and to discuss the pattern of human activities.

### Research methodology

#### TVDI

The temperature vegetation drought index (TVDI) was explicitly proposed by Sandholt et al.^24^ as a method to reflect soil moisture through the wet and dry feature space constructed from the NDVI and surface temperature (Ts). Its calculation formula is as follows:

1 $$TVDI=\left({T}_{s}-{T}_{s-min}\right)/\left({T}_{s-max}-{T}_{s-min}\right)$$

2 $${T}_{s-min}={a}_{1}+{b}_{1}NDVI$$

3 $${T}_{s-max}={a}_{2}+{b}_{2}NDVI$$

where *T*_*s*_ is the surface temperature; *T*_*s-min*_ and *T*_*s-max*_ denote the minimum and maximum surface temperatures corresponding to the NDVI, respectively; and ${a}_{1}$, ${a}_{2}$, ${b}_{1}$, and ${b}_{2}$ are the wet and dry side fitting coefficients, respectively. When the TVDI is between 0 and 1, a larger TVDI indicates more severe drought, and vice versa. According to reference^46^, the TVDI can be categorized into five classes: 0 < TVDI ≤ 0.2, wet; 0.2 < TVDI ≤ 0.4, suitable; 0.4 < TVDI ≤ 0.6, mild drought; 0.6 < TVDI ≤ 0.8, moderate drought; and 0.8 < TVDI ≤ 1.0, severe drought.

#### Trend analysis

The Theil-Sen median method, also known as Sen slope estimation, is a robust nonparametric statistical method of trend calculation^47^, and this method does not require the data to obey a certain distribution and has a strong ability to avoid data errors^48^. Because of the randomness of agricultural drought and the uncertainty of the TVDI time series distribution, the SEN trend method was used to analyse the TVDI change trend in the 20a South China Karst watershed. The calculation formula is as follows^49^:

4 $$\beta =Median\left(\frac{{x}_{j}-{x}_{i}}{j-i}\right)\forall j>i$$

where: median() represents the median value, *j* and *i* are the ordinal time series numbers. A β greater than zero; indicates that the TVDI has an enhancing trend.

The Mann–Kendall (MK) test is a nonparametric test for trends in time series does not require measurements to obey a certain distribution, is not affected by missing values or outliers^50^, and is suitable for significant tests of trends in long time series data^51^.

A trend test is performed using the test statistic Z. The Z value is calculated as follows

5 $$Z=\left\{\begin{array}{c}\frac{s}{\sqrt{Var\left(S\right)}} (S>0)\\ 0 \left(S=0\right)\\ \frac{s+1}{\sqrt{Var\left(S\right)}} \left(S<0\right)\end{array}\right.$$

where: var(S) is the variance, and S is the magnitude of TVDI_*i*_ in relation to TVDI_*j*_ (equation omitted).

In this paper, given a significance level of a = 0.05, the critical value of Z1 − a/2 = ± 1.96. The method of determining the significance of the trend is shown in Table 1.

**Table 1:** Mann–Kendall test trend categories.

$\beta$	$Z$	Trend characteristics
$\beta >0$	$Z>1.96$	Significantly increased
	$Z\le 1.96$	Slightly increased
$\beta <0$	$Z>1.96$	Significantly relieved
	$Z\le 1.96$	Slightly relieved

#### Geoprobe

Geodetector, proposed by Wang Jinfeng^52^, is a tool that can be used to detect and reveal spatial dissimilarity between data, and its model is expressed as follows:

6 $$q=1-\frac{\sum_{h=1}^{L}{N}_{h}{\sigma }_{h}^{2}}{N{\sigma }^{2}}$$

where: *h* is the range of particular explanatory data;* N*; and* N*_*h*_ are the total amount of overall data and the amount of a particular type of explanatory data, respectively; and *σ*; and *σ*_*h*_ are the standard deviations of the overall data and a particular type of explanatory factor, respectively. *q* takes a value from 0 to 1, with larger values indicating that the explanatory factor is more strongly driven by the study variables.

## Results and analysis

### Temporal and spatial patterns of the TVDI in the karst basin of southern China from 2001 to 2020

#### Temporal pattern of the TVDI in the karst basin of southern China from 2001 to 2020

**Figure 2:**
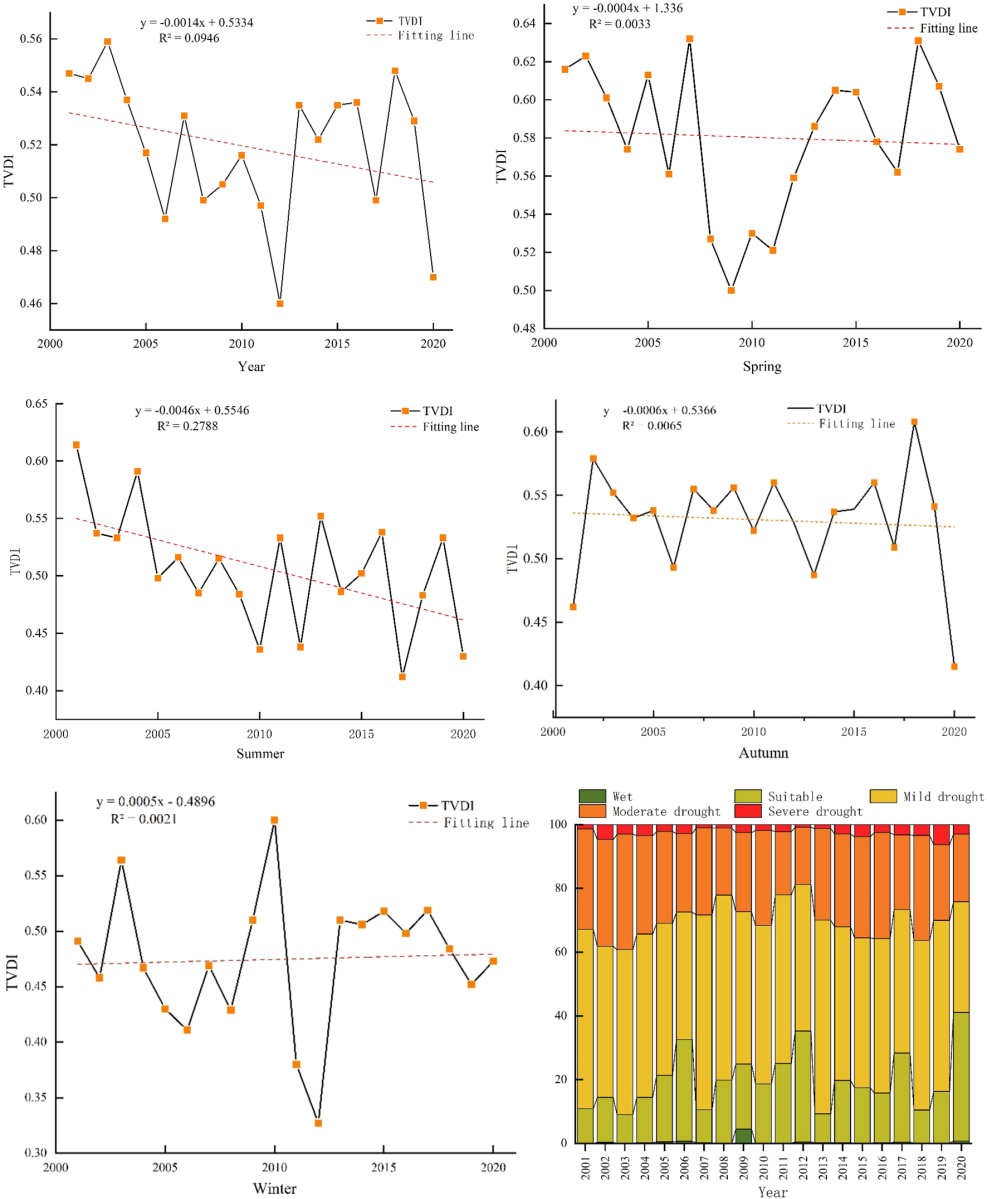

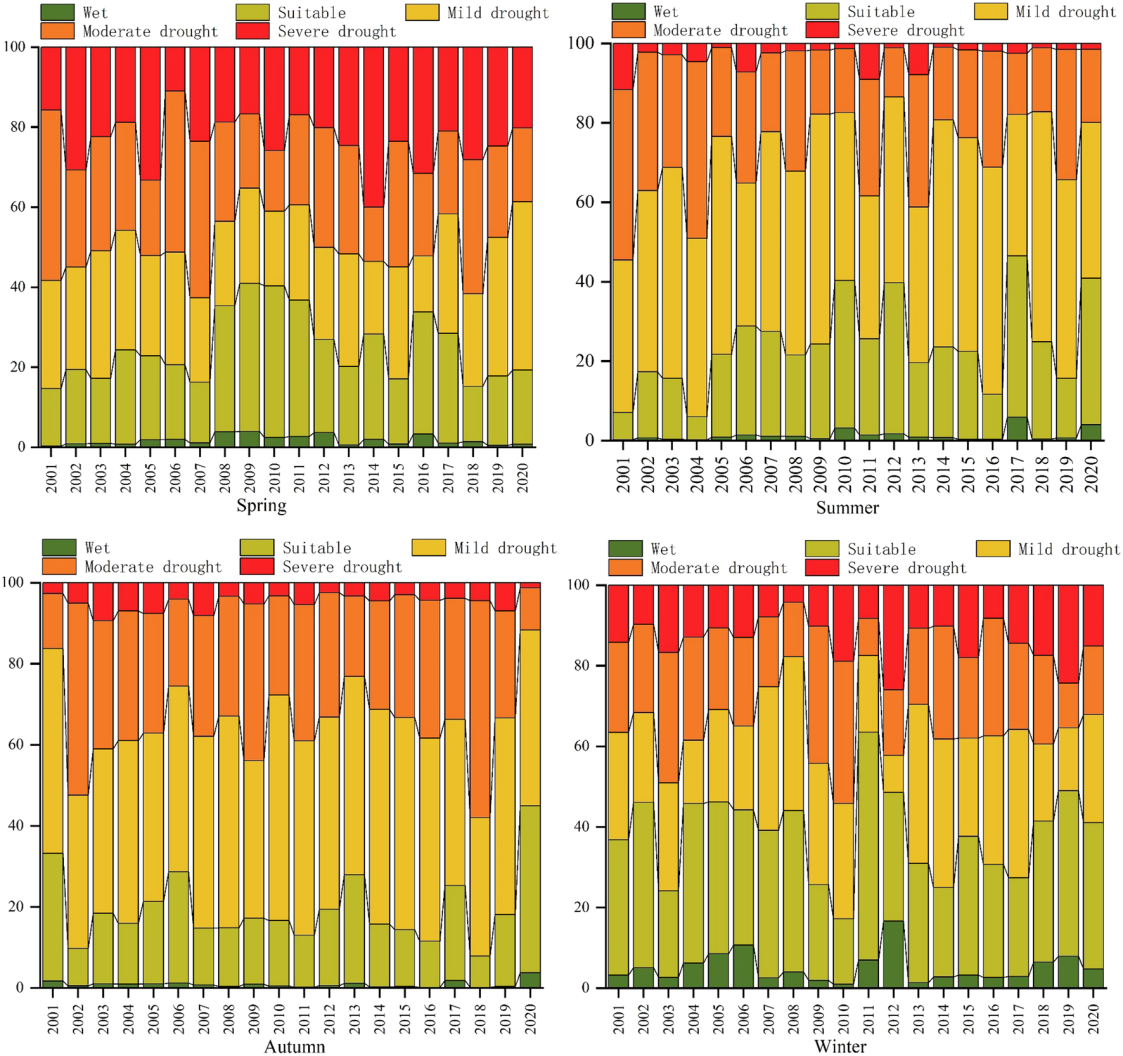
Interannual and seasonal trends in the TVDI and drought area ratio from 2001 to 2020.

Figure 2 shows the interannual and seasonal drought area distributions and change tendencies in the karst basin in southern China. The results showed that the interannual drought index (TVDI) fluctuated and oscillated at a rate of 0.014/10a, with the peak occurring in 2003 (0.56) and the trough occurring in 2012 (0.46). The interannual variation in light drought accounted for the largest proportion (202,292.58 km^2^), followed by that in medium drought (94,591.05 km^2^), and the smallest proportion was in mild drought (1.01 km^2^) (Fig. 2, Table 2). The TVDI decreased at decreasing rates of 0.004/10a, 0.046/10a, and 0.006/10a, respectively, in the spring, summer, and fall (Fig. 2). The largest proportion of drought area in spring, summer and autumn was in 2001, 2004 and 2018, and the smallest was in 2009, 2017 and 2020, with the largest proportion of moderate drought area in spring, summer and autumn. The largest proportion of the area experienced moderate drought in spring and mild drought in summer and autumn (Fig. 2). Compared with spring, summer and autumn, the winter TVDI showed an increasing trend (0.005/10 a), with the maximum occurring in 2010 (0.6) and the minimum occurring in 2012 (0.33) (Fig. 2, Witer). The largest percentage of winter drought area was in 2010, and the smallest percentage was in 2011; the drought class was predominantly suitable, followed by mild drought and the smallest wet class. In summary, the karst basin in southern China from 2001 to 2020 showed no significant interannual changes or seasonal differences within the same drought class (P > 0.05). However, there were significant interannual and seasonal differences within different drought classes (P < 0.001), particularly between different seasons and drought classes (F = 30.2, P < 0.001).

**Table 2:** The interannual and seasonal degrees of drought in different drought grades.

Drought levels	Wet (km^2^)	Suitable (km^2^)	Mild drought (km^2^)	Moderate drought (km^2^)	Severe drought (km^2^)
Year	1.01	49,568.55	202,292.58	94,591.05	6072.81
Spring	297.44	91,097.25	100,240.46	85,462.54	75,428.31
Summer	290.12	47,240.21	237,692.43	66,842.92	460.32
Autumn	29.84	41,672.54	211,917.62	94,950.32	3955.67
Winter	21,151.56	133,959.88	105,757.80	56,404.16	35,252.60

#### Spatial pattern of the TVDI index in the karst basin of southern China from 2001 to 2020

**Figure 3:**
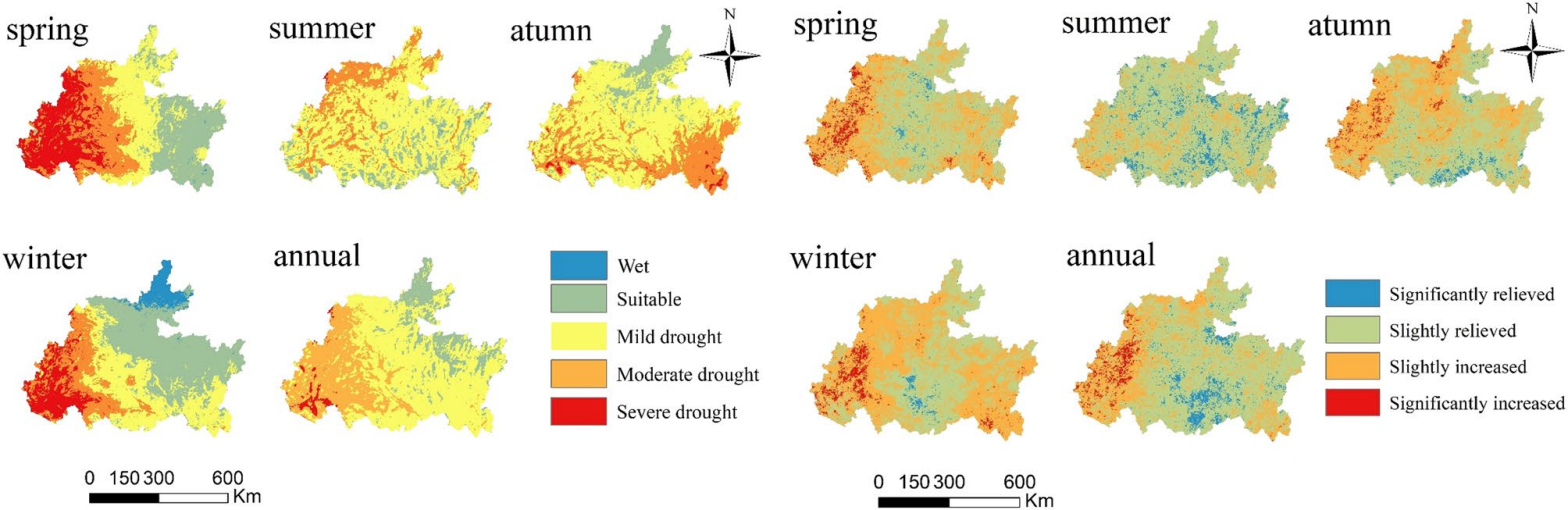
Spatial distribution (left) and trend (right) of annual average and seasonal drought in the TVDI, 2001–2020.The map was created with ArcGIS version 10.2 (https://www.arcgis.com/).

The spatial distributions of the annual mean and seasonal changes in the TVDI in the South China karst basin from 2001 to 2020 are shown in Figs. 3(left). The annual mean and seasonal changes in the TVDI in the South China Karst Basin from 2001 to 2020 were as follows: spring (0.58) > annual mean (0.53) = autumn (0.53) > summer (0.5) > winter (0.48). The spatial distribution of the annual average, spring and winter droughts was "high in the west and low in the east", and the annual average and spring droughts were dominated by mild droughts, while the winter drought was dominated by suitable. Summer droughts decreased from northwest to southeast, autumn droughts gradually decreased from southeast and southwest to north, and mild droughts were dominant in the summer and autumn.

To reveal the evolution of agricultural drought in the karst basin in southern China, in this paper we analysed the trend of annual and seasonal changes in the TVDI using the Theil–Sen median and Mann–Kendall methods (Fig. 3 (right)). The results showed that the annual variation in the TVDI was "significantly increasing in the west and decreasing in the south"; the seasonal variation was "significantly increasing in the west and decreasing in the centre" in spring (Fig. 3, Spring) and "significantly increasing in the west and decreasing in the south" in winter (Fig. 3, Winter). The trend was "decreasing in the south and increasing in the west" in autumn (Fig. 3, Autumn), with a predominantly decreasing trend in summer (Fig. 3, Summer).

### Analysis of influence factors

#### Natural factors

**Figure 4:**
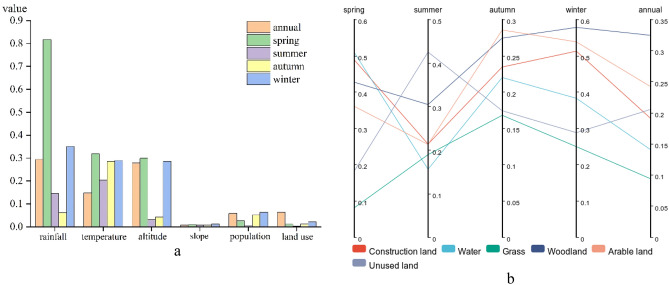
Interannual and seasonal scale one-factor detection (**a**) and spatial heterogeneity in the probability of drought occurrence across land use types (**b**).

To reveal the mechanism of agricultural drought in the karst basin, correlation analyses of natural factors (climate, topography), human activities and the TVDI in the basin were conducted (Table 3), and probes were used to determine the factors affecting agricultural drought (Fig. 4a). Overall, rainfall, temperature, and elevation were most significantly correlated with the TVDI (P < 0.01), at both annual and seasonal scales. The spring rainfall correlation coefficient was R = − 0.812 and that of altitude was 0.458. This was followed by the slope (except at the annual scale, P > 0.05), and the population density correlation did not pass the significance test (except at the annual scale, P < 0.01). Rainfall, air temperature, and elevation were the strongest drivers of agricultural drought, with drivers ranked as follows: rainfall (q = 0.817) > temperature (q = 0.319) > altitude (q = 0.299) (Fig. 4b). At the annual scale, the factors driving the TVDI were as follows: rainfall (q = 0.294) > altitude (q = 0.278) > temperature (q = 0.148) > land use (q = 0.063) > population density (q = 0.059) > slope (q = 0.008). At the seasonal scale, rainfall drove the TVDI the most and slope the least in the spring and winter, and the summer and autumn temperatures drove the TVDI the most.

**Table 3:** Correlation coefficients between the TVDI and impact factors at different scales.

Impact factor	Rainfall	Temperature	Altitude	Slope	Population	Land use
Annual	− 0.404**	− 0.207**	0.458**	− 0.030	0.087**	− 0.018
Spring	− 0.812**	− 0.284**	− 0.365**	− 0.054**	0.018	0.071**
Summer	− 0.212**	− 0.245**	− 0.076**	0.042**	0.017	0.006
Autumn	0.056**	0.438**	− 0.191**	− 0.055**	− 0.025	0.046**
Winter	− 0.372**	0.304**	− 0.442**	− 0.093**	− 0.021	0.075**

"**" is significantly correlated at the 0.01 level.

**Is significantly correlated at the 0.01 level.

#### Human activities

**Figure 5:**
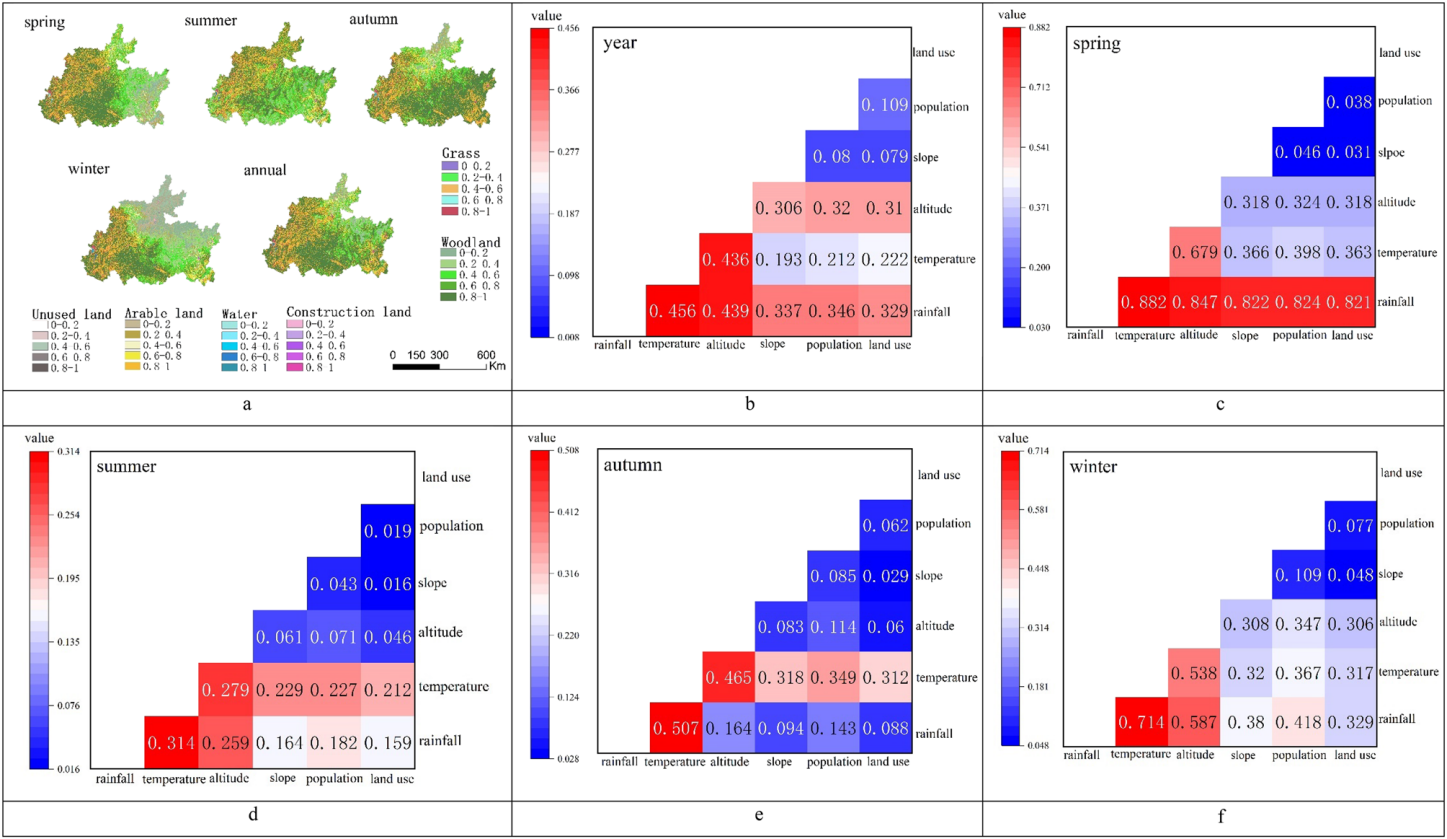
Probability of drought occurrence under different land use types and detection of two-factor driving forces at different scales (**b**–**f**). Maps (**a**) were created with ArcGIS version 10.2 (https://www.arcgis.com/), and maps (**b**–**f**) were created with Origin version 2021 (https://www.originlab.com/).

To reveal the impact of human activities on agricultural drought, we discussed the land use types that drive agricultural drought using conditional probabilities (Fig. 5). Overall, the effects of the annual-seasonal scale and anthropogenic factors on drought were particularly significant in the karst basin in southern China (F = 2.76, P < 0.05, F = 2.99, P < 0.05), and the probabilities of drought (average value) occurrence were as follows: annual (0.86) > autumn (0.84) > summer (0.77) > spring (0.76) > winter (0.68), grassland (0.92) > water (0.8) > cultivated land (0.78) > construction land (0.77) > forestland (0.72) > unused land (0.7) (Fig. 5a). In terms of spatial distribution, the annual, autumn and winter drought probabilities in the karst basin in southern China showed a gradual decrease from southwest to northeast, a "west-high-east-low" distribution in spring, and an increasing trend from southeast to northwest in summer (Fig. 5a). In terms of interannual variation, the difference in the impact of different land use types on drought was relatively small (*Cv* = 0.2), with the largest difference in the impact of forestland (*Cv* = 0.32) and the smallest difference in that of grassland (*Cv* = 0.09). At the seasonal scale, the difference in the impact of different land use types on drought varied from large to small as follows: winter (*Cv* = 0.43) > spring (*Cv* = 0.34) > summer (*Cv* = 0.25) > autumn (*Cv* = 0.23). This was especially prominent in spring (*Cv* = 0.25) > autumn (*Cv* = 0.23), in spring waters, with *Cv* > 0.5 for winter woodland, built-up land and cropland, spring built-up land, woodland and summer unused land (0.4 < *Cv* < 0.5). It was lowest for spring grassland (*Cv* < 0.1).

#### Coupling factors

Since drought occurrence is influenced by multiple factors, we used the geodetector interaction function to explore the interaction effect of different factors on agricultural drought (Fig. 5b–f). At the annual scale compared with the seasonal scale, the interaction factors affecting drought, from largest to smallest, were as follows: spring (q = 0.572) > winter (q = 0.428) > annual average (q = 0.324) > autumn (q = 0.254) > summer (q = 0.191). At the annual scale, the rainfall-temperature interaction factor affected agricultural drought the most (q = 0.456), followed by the rainfall-elevation interaction factor (q = 0.439), and the slope-land interaction driver had the least effect (q = 0.079). At the seasonal scale, the four season rainfall-temperature interaction affected drought the most (up to 0.882), followed by the rainfall-elevation interaction (except for the temperature-elevation interaction in autumn), and the slope-land use interaction had the smallest effect. The single factors that interacted with the remaining factors to drive drought were as follows: rainfall (q = 0.424) > temperature (q = 0.340) > altitude (q = 0.219) > land use (q = 0.188) > population density (q = 0.061) > slope (q = 0.057). Overall, climate change affected drought the most, followed by watershed characteristics, and human activities affected drought to a lesser extent.

## Discussion

In this study, we calculated the TVDI of the Karst Basin in southern China from 2001 to 2020 based on NDVI and LST data and analysed the spatial and temporal evolution of drought in the study area as well as the driving mechanism. The results of this study can better reflect the overall spatial and temporal characteristics of drought in southern China, the future trend of drought change, and the combined effects of natural factors (temperature, rainfall, altitude, slope) and human activities (land use, population) on drought and can systematically reveal the characteristics of the spatial and temporal evolution of drought in the karst basin in southern China and the driving mechanism of each factor. The results of the study can provide a reference basis for drought relief and prevention of droughts and droughts in the karst region.

This study revealed that the TVDI in the karst basin in southern China generally decreased from 2001 to 2020, except for an increasing trend in the western region, and a weakening trend in other regions, which is consistent with the findings of Chen et al.^53,54^. He^44^ used the SPI to analyse the mechanism of hydrological drought in karst watersheds, and the results showed that karst watersheds have a strong water storage function and karst watershed lithology due to a certain degree of solubility. In terms of the differential dissolution and erosion of soluble water, karst watersheds form the most storage space, followed by semikarstic watersheds and nonkarstic watersheds, which in turn suggests that watersheds have a weak-to-strong storage capacity for water: nonkarstic watersheds < semikarstic watersheds < karstic watersheds. This further demonstrates that the causes of agricultural drought in karst basins do not coincide with those in nonkarst basins.

Among natural factors, climate is one of the main drivers of agricultural drought. Table 3 shows that precipitation, air temperature and elevation have positive or negative effects on the TVDI at different time scales. This result may be due to the higher air temperature. A greater difference in air saturation results in more water molecules in the air, which results in a stronger drought regulation ability. The higher the watershed elevation is, the deeper the watershed erosion datum or erosion datum is buried; this results in a greater vertical distance from the watershed surface to the erosion or erosion datum, greater watershed thickness, greater watershed storage space, greater watershed water storage capacity, and greater drought inhibition^55,56^. A steeper watershed slope results in greater rainfall surface runoff, and thus, this scenario has a greater impact on drought; however, a lower watershed slope results in slower rainfall surface runoff, a higher rainfall infiltration rate, more water storage in the watershed, and a stronger ability of watershed water storage to regulate drought^57,58^. Rainfall (q = 0.334), temperature (q = 0.249), and elevation (q = 0.187) were the strongest drivers of agricultural drought, and they were the main factors affecting the occurrence of drought, which was consistent with the conclusions of Zhang^59,60^ and others. Liu^61^ used SSI to explore agricultural drought driving mechanisms in the Fuhe River basin in a nonkarstic region, and the results showed that temperature, solar radiation and wind speed were the strongest drivers of agricultural drought. Xu^62^ studied the response of runoff to climate change and human activities in a karst region. The results showed that the effect of temperature on runoff is much smaller than the effect of precipitation and human activities, contrary to the present study in which the driving force of temperature on agricultural drought is much larger than the factor of human activities. It is possible that temperature has less of an effect on the evaporation of the river within a short period, whereas the environment of a karst region is more fragile, and the change in temperature once it occurs all has an impact on agricultural drought.

The impact of human activities on agricultural drought in karst areas cannot be ignored. The higher the population density is, the stronger the reconstruction and destruction of the surface by human activities, and the stronger the inhibition or promotion of drought by human activities^63,64^. Land use is the way in which human activities act on the watershed medium, and the spatial pattern of land use is the result of human activities on the watershed medium, or land use/cover is the final expression of human activities, and the land use changes in different karstic areas will also affect agricultural drought^65–69^; his suggests that drought is not the result of one factor, and agricultural drought (TVDI) in the karst basin in southern China is strongly influenced by local climate change, and controlled by the spatial distribution of basin topography and geomorphology^70^, as well as by the coupling of local climate change and human activities^71,72^.

In this paper, the NDVI and LST are used to calculate the TVDI monthly; however, the temporal resolution of the NVDI data (16 days) is not consistent with that of the LST data (8 days), whichresults in slight errors in the calculated TVDI. This study is limited by the data of the research stations, which limits applicability of the research results to only a certain region, especially without considering the impact of large-scale atmospheric circulation on agricultural drought. In particular, the effect of large-scale atmospheric circulation on agricultural drought was not considered, resulting in a lack of general applicability of the results of this study. Therefore, the team will further consider the coupling of large-scale atmospheric circulation with local climate change and human activities to drive agricultural drought in future studies, so that the results will be more widely applicable.

## Conclusion

Based on the MODIS data products from 2001 to 2020, the spatial and temporal variations and characteristics of drought in the Karst Basin in southern China in the last 20 a were analysed by calculating the temperature-vegetation drought index (TVDI) with different time scales and spatial variations, and the six influencing factors (temperature, rainfall, land-use type, population density, elevation, and slope) of the TVDI were analysed by using single-factor analysis in Geo-Probe and interactive probing analysis. The following conclusions were drawn:

1. The spatial distribution of the TVDI in the karst basin in southern China has obvious regional characteristics, showing a decreasing trend from west to east, with an increasing trend of drought intensity in the western region (P < 0.05) and an overall spatial trend of "drying in the west and wetting in the south".
2. According to the interannual trend of drought, the degree of drought in the karst basin in southern China weakened over the past 20 a, and drought was most severe in 2003. In terms of the seasonal change in the TVDI, drought in spring, summer and autumn showed a decreasing trend, drought in winter showed an increasing trend, with significant differences in the spatial distribution of drought, and the overall change in drought was seasonal. The drought intensity decreased in the following order: spring (0.58) > autumn (0.53) > summer (0.5) > winter (0.48).
3. The results of single-factor detection showed that rainfall, temperature and elevation were the main factors driving the formation of aridification in the study area; multifactor coupling drove drought in descending order: rainfall (q = 0.424) > temperature (q = 0.340) > elevation (q = 0.219) > land use (q = 0.188) > population density (q = 0.061) > slope (q = 0.057). Therefore, the occurrence of drought is the result of a combination of factors, especially rainfall, which is the most important factor influencing the generation of agricultural drought.

## Data Availability

All the data generated or analysed during this study are included in this published article.
